# Siloxanes in Biogas: Approaches of Sampling Procedure and GC-MS Method Determination

**DOI:** 10.3390/molecules26071953

**Published:** 2021-03-30

**Authors:** Grzegorz Piechota

**Affiliations:** GP CHEM, Laboratory of Biogas Research and Analysis, Legionów 40a/3, 87-100 Toruń, Poland; gp@gpchem.pl

**Keywords:** siloxanes, biogas, biomethane, GC-MS, sampling

## Abstract

A new approach of siloxane sampling based on impinger, micro-impinger, adsorption on active carbon, and direct TedlarBag methods followed by gas chromatography-mass spectrometry (GC-MS) was developed for the analysis of three linear (L2–L4) and four cyclic (D3–D5) volatile methyl siloxanes (VMSs). Three kinds of organic liquid-medium characterized by different polarities, namely acetone, methanol, and d-decane as siloxanes trap were arranged in the experiment which is widely discussed below. Thus, the GC-MS equipped with SUPELCOWAX-10 capillary column was employed to perform monitoring of VMS content in the analyzed biogas samples originating from landfill, wastewater treatment plants, and agriculture biogas plants. In all samples that have undergone the analysis, cyclic and linear VMSs were found in quantities exceeding 107.9 and 3.8 mg/m^3^, respectively. Significant differences between siloxanes concentrations depending on biogas origin were observed. Moreover, the high range of linearity (0.1 to 70.06 mg/m^3^), low LoD (0.01 mg/m^3^), low LoQ (0.04 mg/m^3^), and high recovery (244.1%) indicate that the procedure and can be applied in sensitive analyses of silica biogas contaminants. In addition to the above, the impinger method of sampling performed better than active-carbon Tube and TedlarBag, particularly for quantifying low concentrations of siloxanes. Overall, the evaluation of sampling methods for biogas collection simplified the analytical procedure by reducing the procedural steps, avoiding the use of solvents, as well as demonstrated its applicability for the testing of biogas quality.

## 1. Introduction

Regarding the reduction of the emission and continuously increasing demand for renewable resources, the concept of biogas [1,2] and biomethane utilization [3] have gained a special significance. During the last 10 years, studies on biogas and biomethane and the determination of silica compounds in the form of volatile methylsiloxanes (VMSs) has been investigated [4,5,6]. Moreover, green chemistry, including biogas analysis as well as green technologies in the form of biogas purification require simple and rapid analytical methods for the evaluation of their environmental impact. Thus, the green techniques and methods constitute an important issue in the modern analytical procedure. The analysis of siloxanes in different types of biogas and high energetic samples is a challenging task. In recent years, different studies have pointed out that siloxanes presented in biogas matrices have effects on biogas motors installed in combined heat and power units (CHP) [7]. Moreover, their presence is responsible for the shortening of engine lifetime and the increase in operational cost [8].

Thus, the siloxanes involve a large group of chemical compounds with molecular weights from a few hundred to several thousand, however, the silica-contaminants existing in biogas are man-made compounds containing silicon and oxygen with organic side groups (methyl groups) attached to the silicon atoms and are called methylsiloxanes. This special Si–O bond imparts the unique properties of siloxanes. Siloxanes free energies were estimated from bond energies in order to calculate the strength during bond dissolution. The values of siloxanes free energies estimated from bond energies is listed in Table 1. 

According to IUPAC nomenclature, methylsiloxanes are characterized by the linear and cyclic structural configurations (designated by L and D for linear and cyclic form, respectively). Furthermore, the commonly appearing siloxanes in biogas originating from different sources are linear hexamethyldisiloxane (abbreviated as L2), octamethyltrisiloxane (L3) decamethyltetrasiloxane (L4) and cyclic hexamethylcyclotrisiloxane (D3), octamethylcyclotetrasioxane (D4), decamethylcyclopentasiloxane (D5), and dodecamethylcycloheksasiloxane (D6). It should be noted that the low Henrys law constant combined with low water solubility suggests that VMSs have a strong tendency to be volatilized and both can appear in the air and could be detected in the landfill area. In addition to the aforementioned, in saturated water, other silica-contaminants of biomethane in the form of trimethylsilanol (abbreviated as TMSOH or TMS) can occur. Due to the –OH group bonded directly to an Si atom, the TMSOH has good solubility in water. In contrast to trimethylsialnol, as presented in VMSs structure, organic methyl groups bonded with an Si atom give siloxanes strong hydrophobic properties such as low solubility, thermal stability, and hydrophobicity [10]. The physical and chemical properties of VMSs are listed in Table 2.

The combustion of biogas contained VMSs in CHP unites results in the formation of silica-deposit inside the motor. The deposit is formed depending on the temperature and pressure, while oxygen availability can shape various morphological from strong and crystalline to amorphous deposits that are associated with other elements such as sulfur or calcium. 

This accumulation affects the engine performance by decreasing the efficiency of biogas conversion to energy. In the reactions below, the chemical reactions of VMSs oxidation (linear and cyclic VMSs) are presented, where the final product is in the form of silicon oxide (IV).

1. C_8_Si_3_O_2_H_24_ + 16 O_2_ → 3 SiO_2_ + 8 CO_2_ + 12 H_2_O (linear VMS—L3)
2. C_10_Si_5_O_5_H_30_ + 20 O_2_ → 5 SiO_2_ + 10 CO_2_ + 15 H_2_O (cyclic VMS—D5)

In the context of biogas upgrading to biomethane and siloxanes impact on CHP units, it is important to understand siloxane oxidation and crystal deposit formation. At first, under extreme conditions (high temperature and pressure in CHP units) the silica components present in the biomethane convert to the liquid amorphous glass form. After CHP unit cooling, the amorphous phase crystallizes and forms a layer of micro-glass deposit. This micro-crystal silica deposit covers the combustion chamber, causing a higher emission of air pollutants and repeated violation of air emission regulations [8]. Likewise, high concentrations of the discussed compounds lead to the overheating of sensitive motor parts and to the malfunction of pistons and spark plugs in biogas motors. Furthermore, the silica atoms could be observed in the engine oil [8] and gas grid biomethane stream. Therefore, the controlling of VMSs concentration in biomethane and their removal prior to injection into the gas-grid system is an important issue for gas-grid operators [11].

The main objective of the present study was focused on how to determine VMSs in biogas samples originating from different sources, with the use of various sampling methods together with applying a gas chromatography technique coupled with a mass spectrometry detector (GC-MS). Additionally, use of four different biogas sampling methods and organic solvents with a wide range of polarities created a real perspective of a rapid method for biogas silica-contaminants quantification and allowed for the monitoring of siloxanes concentrations by an online system.

To the best of our knowledge, there are no other works that extensively discuss the sampling methods of siloxanes, the differences in siloxanes concentrations that result from the use of trapping solvent with the impinger method, and the origin of biogas (landfill, WWTP, and agriculture biogas plants). Moreover, in contrast to the existing research, this study reveals the examination of four sampling methods as well as calculates the recovery for each determined siloxane resulting from applied sampling methods. The study represents a proof of concept that is a consecutive step in the development of real operated installations biogas sampling and its sensitive analysis by the GC-MS technique. The presented experiments allowed us to understand that the proper combination of sampling method and applying solvent in sensitive siloxanes analyses can compete against other methods based on solid phase extraction in terms of time, cost, and simplicity.

## 2. Results

It must be emphasized that the development of methods for siloxanes determination in biogas required the use of modern equipment and new technique of biogas collection performance. The qualitative and quantitative determination of linear and cyclic siloxanes was achieved by applying a gas chromatography technique coupled with a mass spectrometry detector. In the procedure, the use of the following three solvents: acetone, methanol, and d-decane was tested. Likewise, the GC-MS technique was applied for the determination of VMSs concentration in biogas sampled in four different ways (with impinger, micro-impinger, with AC sampling tube, and direct from TedlarBag). The comparison of different concentrations of VMSs in analyzed samples allowed to describe and characterize biogas depending on its origin.

Consequently, for all analyzed biogases, some significant differences in the concentration of siloxanes were observed. Providing a general statement, the differences were observed between the concentration of linear and cyclic siloxanes. Moreover, the siloxane concentrations strongly depended on the sampling procedure. In the performed study, all cases of biogas siloxanes were detected and quantified. Therefore, in all analyzed samples the linear and cyclic forms of siloxanes were observed. Accordingly, the concentration of siloxanes in analyzed samples of biogas was observed in the range from 71.6 to 112.0 mg/m^3^, from 14.9 to 30.2 mg/m^3^, and from 0.3 to 1.9 mg/m^3^ for landfill gas, WWTP biogas, and agriculture biogas, respectively. The results of the experiment including different types of used solvents and sampling procedures are listed in Table 3.

### 2.1. Procedure Validation

In order to define the repeatability of the VMSs determination method, six replicates of GC-MS shots were analyzed and the relative standards deviation (RSD) was calculated. Repeatability of the chromatographic determination was determined by injecting six times standard solutions of 20 µg g^−1^ for linear siloxanes and of 70 µg g^−1^ for cyclic VMSs with an automatic injector, as well as manually into the GC-MS system. Additionally, the range of linearity for each calibration curve was calculated. Finally, the sensitivity of the methods was examined. With respect to the complexity of the samples, the limits of quantification (LoQ) and limits of detections (LoD) were evaluated for each VMSs, separately. Consequently, recovery experiments were carried out for all sampling methods. The recovery that was carried out for each sample depended on the method of sampling. The GC-MS parameters including calibration curves and validation parameters are listed in Table 4. 

**Figure 1:**
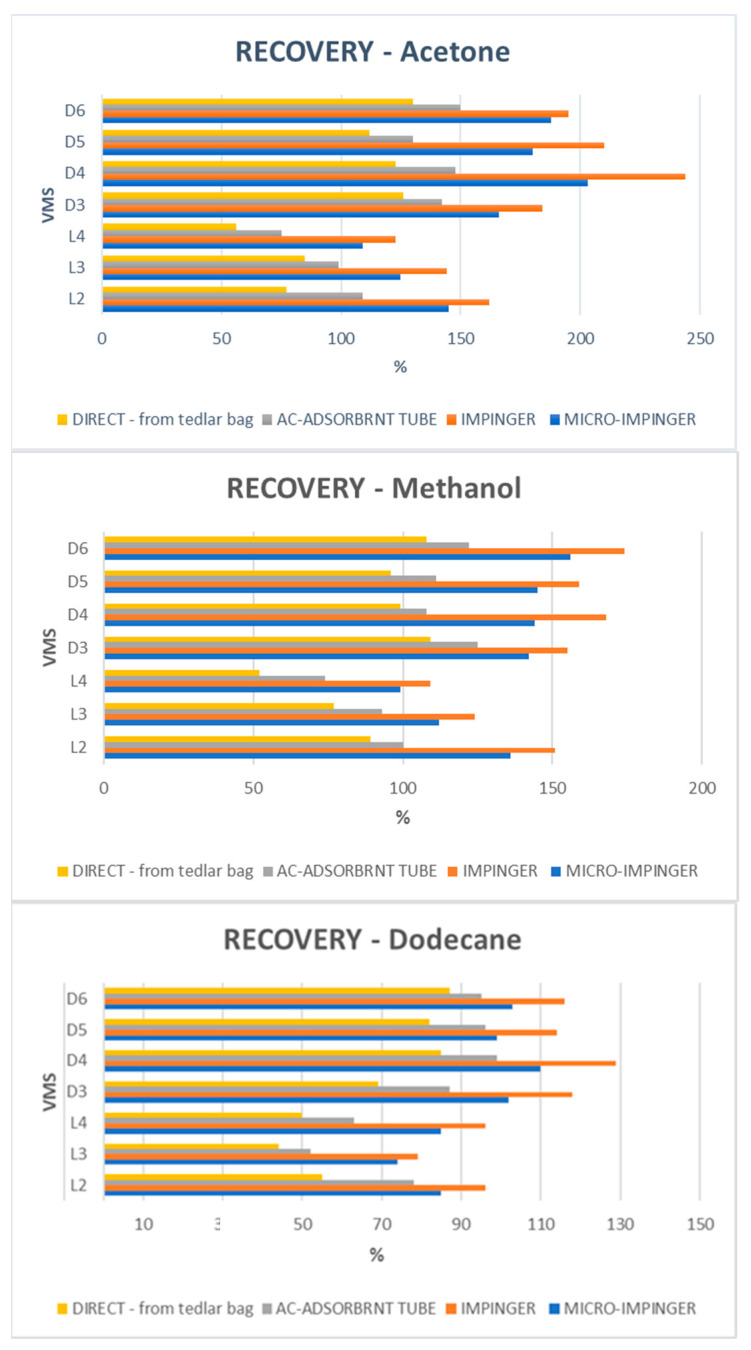
Recoveries of siloxanes from biogas depended on the sampling procedure and the solvent used.

The experiments of recovery were carried out by spiking biogas samples at three levels of each siloxane: 1, 5, and 20 mg/m^3^. The fortified samples were kept at room temperature for two days to allow the siloxanes to volatilize into the biogas sample. Unspiked “blank” samples were analyzed as a real concentration of silica compounds in the analyzed type of biogas. As a next step, the recoveries were calculated by dividing the difference between the measured concentration of VMSs for spiked and unspiked samples. The findings revealed that recoveries for linear and cycling VMSs ranged between 44.2 and 162.6% and 69.7 and 244.1%, respectively. The recoveries for all analyzed siloxanes are shown in Figure 1.

The performance of repeatability, of the entire analytical procedure was determined by analyzing five biogas samples spiked with 1 mg/m^3^ of each siloxane. The relative standard deviation (RDS) was obtained in the range from 2.78 to 5.68%, from 0.58 to 8.14%, and from 3.84 to 9.52% for landfill biogas, sewage biogas, and agriculture biogas with the application of acetone, respectively. The repeatability of the chromatographic determination was determined by injection three times of standard solutions (as the mixture of VMSs in acetone) with the automatic injector. The concentration of siloxanes in standard solution was prepared as follows: L2, L3, L4: 1 µg mL^−3^, D3: 3 µg mL^−3^, D4, D5: 15 µg mL^−3^, and D6: 1 µg mL^−3^ in a 25 mL glass vial.

Following, the linearity was studied by performing a six-point calibration curve in the levels expected in biogas samples according to its origin. As presented in Table 4, the range of linearity for the applied GC-MS method obtained in the measurements varied in the range of 0.11 to 25.16 mg/m^3^ for linear and from 0.10 to 70.06 mg/m^3^ for cyclic siloxanes. Moreover, the correlation coefficients for all prepared calibration curves equaled from 0.966 to 9.998 and from 0.991 to 0.999 for linear and cyclic siloxanes, respectively.

Consequently, the parameters in the form of LoD and LoQ were calculated. The calculation was conducted considering a signal-to-noise ratio of 3 and 10, respectively, using the level of the calibration curve prepared in acetone. In addition, no siloxanes were detected in the blank solutions. The obtained results in the LoD experiment were on significantly low level, from 0.01 to 0.03 mg/m^3^ for the impinger method, and in the range from 0.01 to 0.04 mg/m^3^ for the AC-tube extraction method, and from 0.02 to 0.08 mg/m^3^ for TedlarBag direct injection. Moreover, the LoQ was observed in the range from 0.05 to 0.10 mg/m^3^ and from 0.04 to 0.06 mg/m^3^ for linear and cyclic siloxanes, respectively.

It should be pointed out that the obtained results of LoD and LoQ were the first limits reported for different types of sample collection (micro-impinger, regular impinger, AC-extraction, and direct from TedlarBag), in particular concerning biogas coming from real operated plants including landfill, WWTP, and agriculture biogas. The parameters in that form of RSD, range of linearity, LoD, LoQ, and correlation coefficients for all prepared calibration curves are presented in Table 4.

### 2.2. Gas Chromatographic Siloxanes Determination

Consequently, the chromatographic separation of six siloxanes L2, L3, L4, D3, D4, D5, and D6 was optimized and evaluated. The tested high polarity column allowed to determine siloxanes in all types of biogas that were engaged in the experiment. In order to obtain results from the GC-MS technique, the single ion current monitoring (SIM) mode of the GC-MS was tested and the samples chromatograms were analyzed. Thus, the GC-MS results were obtained with increased accuracy, precision, and repeatability.

#### 2.2.1. Agriculture Biogas

It was revealed that the agriculture biogas was characterized by a very low concentration of siloxanes. Similar values were demonstrated in our previous work [8]. The concentration of siloxanes in agriculture biogas did not exceed 2.0 mg/m^3^ and the linear form of siloxanes was not observed. The siloxanes concentrations were in the range from 1.0 to 1.9 mg/m^3^ and from 0.3 to 0.7 mg/m^3^ for sampling by applying the impinger (with acetone) and tube with AC (extraction solvent: acetone), respectively. For the direct sampling method and for using dodecane as a solvent, no siloxanes in agriculture biogas were detected. In addition to the above findings, in the context of the used solvents, the concentration of siloxanes measured in acetone was examined to be about 81% higher than that quantified in methanol. The differences appearing between the application of the micro-impinger and regular-sized impinger was 21.05% of the value. The obtained results clearly indicate that sensitive analyses from the gas phase required use of a solvent with high polarity.

#### 2.2.2. WWTP Biogas

The biogas from WWTP was tested and siloxanes in the form of L2, D3, D4, D5, and D6 were consequently detected and quantified. The linear siloxanes in the form of L3 and L4 were not detected. In comparison to our previous work [12], L3 and L4 were analyzed using methanol as an organic solvent. The highest concentration was observed in samples where the impinger and micro-impinger were applied—30.2 and 29.2 mg/m^3^, respectively. Moreover, cyclic forms, mainly D4 and D5 were predominant, whereas linear forms represented less than 4.70% of all silica forms presented in the analyzed WWTP biogas samples. The significant differences in siloxanes concentrations were observed in the context of the applied solvent and the procedure of sampling. The concentration of siloxanes in samples collected with the use of the impinger method was higher than determined with the use of AC-tubes and the direct procedure. In accordance with the obtained results, the concentration of siloxanes in samples collected while using the impinger was about 47.01% higher than the concentration found in samples from the direct procedure. On the other hand, the polarity of used solvents played a main role in the adsorption and extraction and it was observed that the polarity of used solvent improved the efficiency of the extraction process. Furthermore, in sewage biogas analyzed direct from the TedlarBag, siloxanes in their linear form were not found.

#### 2.2.3. Landfill Biogas

**Figure 2:**
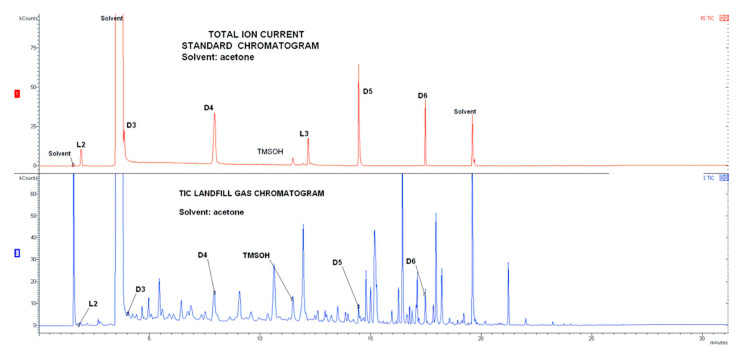
The GC-MS chromatograms (TIC mode) of siloxane standards and organic matrix of landfill biogas.

Contrary to biogas from WWTP and the agriculture biogas plant, the landfill biogas was characterized by the complexity of the biogas organic matrix. The matrix contained significant amounts of organic compounds including terpenes and other cyclic hydrocarbons. Not surprisingly, in the landfill gas, more than 80 organic components were found. Therefore, the matrix effect has a negative impact on the accuracy of the GC-MS results obtained for landfill biogas analyses. The GC-MS chromatogram plot with complex siloxane standards and organic matrix of landfill biogas is presented in Figure 2.

**Figure 3:**
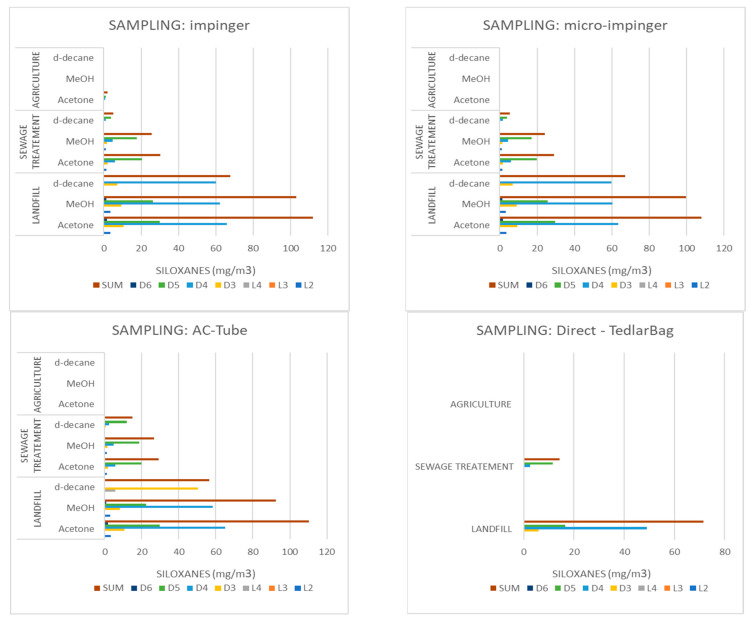
The concentrations of siloxanes in the context of used solvent, sampling procedure, and type of biogas.

In the landfill biogas, all the examined siloxanes were found. Additionally, the concentration of siloxanes in the analyzed samples depended on the type of solvent applied, as well as the procedure of sampling itself. The concentration up to 108.0 mg/m^3^ was determined for the impinger method using acetone as an organic solvent. Moreover, significant differences in the concentration of siloxanes were observed for the impinger method and direct-injection analyses. During the operation of the direct injection from the TedlarBag, no linear siloxanes were found, while D6 remained undetectable. This finding could have referred to the strong “siloxanes wall stick effect” [8]. In the procedure with the AC-tube, applying the L3 and L4 was undetectable, while the difference between the concentration of siloxanes was maintained at a level of about 3.38% and 36.12%, when comparing with the impinger method and direct-sampling, respectively. The detailed concentrations of the examined siloxanes in the context of the used solvent, sampling procedure, and type of biogas are illustrated in Figure 3.

Thus, a considerable variation in the quantity of VMSs in biogas originating from different sources was reported in the lecture [19]. However, new information on siloxane determination involving the application of the GC-MS technique, as well as four different approaches on sampling procedure with simultaneous application of solvents with various polarity was not found. The determination of siloxanes in biogas from anaerobic digestion of wood waste and in biomethane was reported by Ghidotti et al. [4], while the method of solid-phase microextraction was applied, and the linear and cyclic VMSs were detected. The determination of siloxanes in landfill biogas was reported by Vaughan et al., and the applied technique based on GC-MS equipped with SIFT detector [34]. On the other hand, the profile of VMSs was present in the landfill gas and biogas from WWTP as it was described by Tansel et al., [35] and the carbon adsorption tube was tested [36]. As the literature presents, the VMSs and other biogas impurities in the form of sulfur and halogenated compounds were stored short-term. Moreover, the suitability of different adsorbents was examined [37]. As we reported, the gas chromatography technique coupled with an FID detector was applied, however, the internal standards were required by the procedure [9]. Furthermore, in the GC-FID technique, the complex organic matrix of biogas played an important role and the linear form of siloxanes was difficult for quantification [9]. The available new analytical methodology for VMSs and TMSOH determination in biogas samples was reported by Raich-Monitu et al., as well as, the GC-MS in SIM mode with four different columns being reported: HP-5MS; TRBG43; DB1701; SUPELCOWAX-10 [38]. Shweigkofler and Niessner [19] reported the determination of VMSs by applying the GC-ACD method. Obtained results ranged from 0.06 to 0.23 mg/Nm^3^, and from 5.76 to 16.83 mg/m^3^ for linear and cyclic VMSs, respectively.

The concentration of siloxanes in biogas for WWTP processes was reported in the work of Beese et al., where 308 measurements were determined, and an average concentration of VMSs in sewage sludge samples and a concentration of about 15 mg Nm^−3^ (exact: 0 to 317 mg Nm^−3^) were found [39].

The gas chromatography coupled with an MS detector and equipped with an FT-IR multi-component gas analyzer was tested by Arnold and Kajolinna [23] and the reported system was portable and equipped with three columns. Moreover, for siloxanes determination, the gas chromatography technique was coupled with other types of detection: AES [19], PID [23], or was installed in tandem MS-MS with an APCI detector [40]. For the PID detector, as authors have reported, the Supelcowax 10^TM^ and Carbopack^®^B columns were used. Similarly, the concentration of siloxanes in sewage gas from WWTP using the LT-GC/ICP-MS method was in the range of 8.5 to 11.7 mg/Nm^3^ as revealed by Grumping et al. [21] in Germany. On the other hand, the siloxanes concentrations in sewage treatment were determined in several WWTPs in Europe. The presence of siloxanes in cities such as Zurich (Switzerland), Neuburg (Germany), Sint-Triuden (Belgium), and Tricatti (UK) in amounts 25.1, 59.8, 20.0, and even up to 400 mg/Nm^3^ were checked [22]. The cyclic siloxanes in the form of D4 and D5 were found in waste active sludge, where the GC-FID technique and extraction procedure with using n-hexane as an extraction solvent were applied, while the VF-1MS capillary column was applied [20]. In addition, as Bailey [40] reported, for biogas originating from digestion processes, the concentration of siloxanes in the range of 7.7 to 27.3 mg/m^3^ was determined. The GC coupled with AED/MSD was developed for siloxanes analysis [19]. The authors used complex instrumentation for siloxanes quantifications. Therefore, the obtained results showed that the concentration of siloxanes in sewage gas was about 16.54 mg/m^3^, however, the experiment was conducted during the ten days with the use of steel canisters. The recovery at day 10 was in the range of 100 to 106% in relation to the first day of the experiment. Additionally, siloxanes were kept in steel canisters and the “wall stick effect” was not observed, which was confirmed by a high recovery value. Finally, in the lecture there is a review of siloxanes presence in the environment related to the period from 1970 to 2017 in the literature. As Mojsiewicz-Pieńkowska reported, siloxanes were presented in samples of air, water, biogas, and solids [41].

### 2.3. Sampling Approaches

To gain a deeper understanding, a combination of four different sampling approaches and of three organic solvents for absorption of siloxanes were examined. In order to obtain information on sampling approaches and solvent efficiency, the recoveries were calculated and are presented in Figure 3. The results confirmed the highest absorption performance was obtained when the acetone was used as the impinger trapping solvent and solvent for extraction of AC-tubes. The concentrations of siloxanes in tested biogases were found in the range of 108.1, 112.2, 110.30, and 71.6 mg/m^3^ for sampling using the micro-impinger, impinger, AT-tube, and performance of direct injection (TedlarBag), respectively. The results of GC-MS analysis revealed that 100% out of all VMSs were adsorbed in acetone while only 94.4% and 65.8% of them were adsorbed in methanol and d-decane for landfill biogas, respectively. Moreover, the overestimated derivative values of RSD for results from the GC-MS method were an outcome of matrix complexity, particularly its organic composition. The analysis of biogas from sewage sludge showed that 100% of all VMSs were adsorbed in acetone, while only 82.8% and 22.7% out of them were adsorbed in methanol and d-decane, respectively. In the case of agriculture biogas, 100% out of all VMSs were adsorbed in acetone while, only 20.0% out of them were adsorbed in methanol. In d-decane, siloxanes were undetectable. The comparison of the regular size impinger and micro-impinger sampling methods showed that the micro-impinger should not be used for sampling where the concentration of siloxanes in analyzed medium is relatively high (>60 mg/m^3^). On the other hand, the micro-impinger can be applied for agriculture biogas analysis. The comparison of used solvents VMSs absorption efficiencies depended on their polarities. It was visible in all samples that were examined.

### 2.4. Further Routes of Development

The present works analytical procedures and comparison of different approaches of sampling allowed to conclude routes for further development of biogas silica-contaminants analytical methods. The investigation on used solvent range of polarity and solvent affinity to siloxane molecules is a promising route of development of sensitive and rapid tests for siloxanes concentrations for commercial use. The important issue was related to the capillary column used: the fully-saturated GC-MS column is more appropriate for the determination of cyclic siloxanes, than their linear forms. For this reason, research on the separation mechanism inside the column is needed, as well as research on the GC-MS parameters in order to shorten the of analysis time. On the other hand, the described approaches, particularly direct-sampling, require more investigations and experiments to improve the efficiency and sensitivity of siloxanes determination in real and raw biogas streams.

## 3. Materials and Methods

### 3.1. Standards, Reagents, and Calibration Curves Preparation 

In the presented study, the regular-impinger and micro-impinger methods with three kinds of organic solvents (acetone, methanol, and dodecane) were used for the determination of VMSs (incl. L2, L3, L4, D3, D4, D5, D6) from biogas samples. Solvents with purity (99.8 to 99.99%) for gas-chromatography were obtained from SIGMA (Sigma Aldrich, Toruń, Poland). The AC-tube extraction medium in that form of a mixture of acetone/methanol (50/50; *v*/*v*)—SupraSolv with purities for GC analysis (99.98%) was provided by SIGMA (Sigma Aldrich, Toruń, Poland). Then, for direct sample collection, the TedlarBag (2.5 L/PlastiGas, LindeGas, Poland was used. The solutions coming from impingers (regular and micro-scale), after extraction processes of AC-Sampling Tube and direct from the PlastiGas—TedlarBag were measured on the gas chromatography system equipped with the mass-spectrometry detector (GC-MS), respectively. The glassware: impinges, bottles, pipes, and caps used in our study were obtained from Schott Duran (Duran Group, Binovo, Legnica, Poland), whilst the vials and septa with parameters ND-15-PP/Butyl/PTFE for sample storage were provided by NeoLab (NeoLab, Heidelberg, Germany). Both, vials and septa were used as received without any pre-treatment.

The calibration curves were prepared by dissolving VMS standards in acetone, methanol, and dodecane. Since the findings revealed that acetone had the best percentage recovery, the calibration curves for the determination of VMSs concentrations were prepared in acetone. The calibration levels were chosen for VMSs concentrations in the biogas depended on their origin. It was decided for GC-MS that calibration curves would be six-point calibration lines. The parameters of calibration curves for the GC-MS method, limit of detection (LoD), limit of quantification (LoQ), determination coefficient (R^2^), and range of linearity are listed in Table 4.

### 3.2. Biogas Samples and Sample Preparation

Regarding the requirements of the conducted examination, the tested biogas originated from different sources and locations. The biogas samples were collected from operating plants of landfill (located in Świętokrzyskie Voivodeship) municipal waste water treatment (located in Kujawsko-Pomorskie Voivodeship), and agriculture biogas (located in Warmińskio-Mazurskie Voivodeship) in Poland. The biogas was collected in four independent approaches of sampling: direct from TedlarBag, with the use of an active-carbon sampling tube, with impinger and micro-impinger (scale 1:20, *v*/*v*). The sampling procedure was carried out on the spot in all analyzed sites. The absorption of VMSs in liquid solvents was carried out using the real, utilized biogas. In this case, in the main biogas pipe was an up-pressure, therefore, the accuracy pump was not the integral part of the used equipment. During the experiment itself, 20 L of biogas was sampled for each sampling trial. In the investigation, 60 samples were taken.

Then, for direct sample collection, the TedlarBag (2.5 L/PlastiGas, Hamburg, Germany) was tested. The biogas was sampled from the main biogas pipe directly to the TedlarBag, and transported to the laboratory afterward. The biogas was injected into the GC-MS system without any additional procedures.

The sampling that applied an active-carbon sampling tube was carried out on on-the-spot and the determination tests were conducted in the laboratory with a simultaneous extraction procedure. The glass tube with AC was characterized by two areas: test and control with amounts of AC 2.0 g and 0.25 g, respectively (the grain of AC: 2–3 mm). The biogas flow rate was set up in the range of 100 to 250 mL/min. Duration of the performance of sampling varied from 10 to 25 min, with a final max. 20 L of biogas going through the AC-tube. The extraction method was carried out with the application of three organic solvents: acetone, methanol, and dodecane.

**Figure 4:**
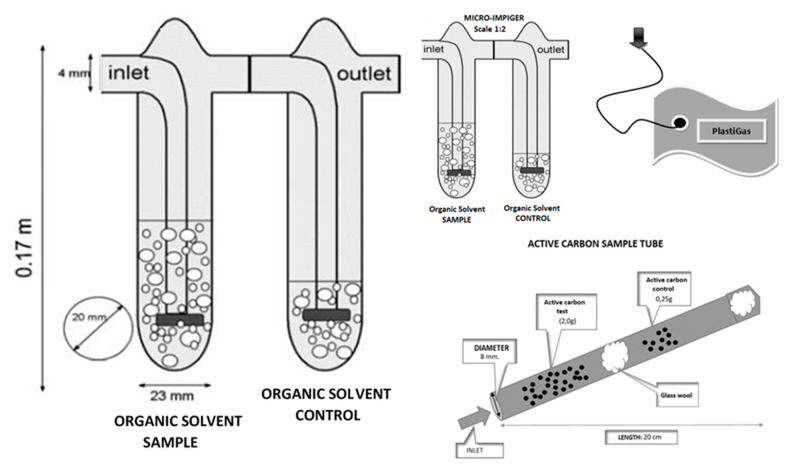
Sampling equipment.

The impinger method (micro-scale and regular-scale) was involved for biogas in the conducted experiment. For the determination of VMSs, the two series-connected impingers were applied. The sample and control contained approximately 30 and 10 g of used solvent (sample and control as a first and second impinger, respectively). Acetone—SupraSolv with purities for GC analysis (99.99%)—was provided by Merck (Merck, Hamburg, Germany). Methanol—SupraSolv with purities for GC analysis (99.97%)—was provided by Merck (Merck, Hamburg, Germany) and Dodecane—POCH with purities for GC analysis (99.98%)—was provided by POCH (POCH, Gliwice, Poland). Consequently, the gas flow rate was set to 0.5 L/min for 40 min and approximately 20 L of biogas went through the sampling system. During the process of sampling and afterward, the solutions from impingers were stored in a cool (<0 °C) environment. The operation of sampling was repeated three times. Finally, 60 samples were taken. Then, the obtained solutions, after absorption processes, were analyzed by the GC system coupled with an MS detector. The micro-impinger was scaled in the ratio: 1:2 (incl. weight of solvent and biogas flow). The sampling equipment is shown in Figure 4.

### 3.3. GC-MS System

**Figure 5:**
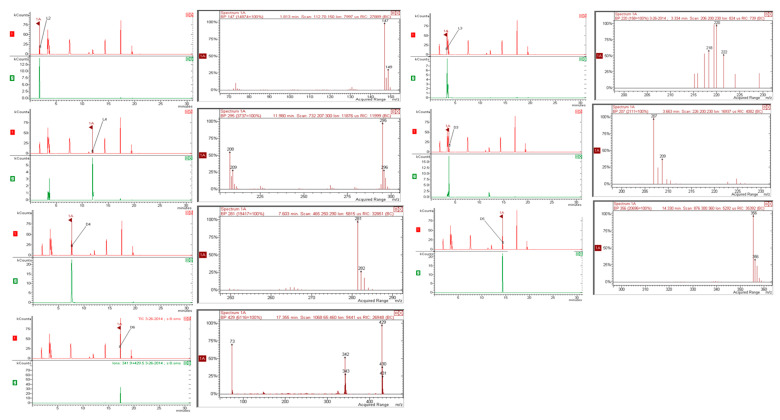
The GC-MS spectra ion fragments used for identification and quantification of siloxanes.

The VMSs were measured using a Varian 3800 gas chromatograph equipped with a CTC autosampler (CTC Pal Autosampler, Germany) and it was coupled with an IT2200 MS detector (Saturn, MS-IT2200, Germany). The mass selective detector was characterized by the temperature of the transfer line at 220 °C. The mass spectrometric detector was operated in electron impact ionization mode with an ionizing energy of max. 80 eV, while the electron multiplier was operated at 1200 V. The dynode voltage was set as 6 kV. The selected mode and the mass window were set to ion monitoring (SIM) and in the range from 15 to 500 *m/z*, respectively. Additionally, the GC-MS conditions were as follows: the initial oven temperature was 70 °C and the initial ramping rate was 5 °C min^−1^ to 200 °C with subsequent sample conditioning at 60 °C with a duration of 10 min isothermal conditioning. Optional parameters of the system: injector temperature, 240 °C, detector temperature, 230 °C. Carrier gas: helium (linear gas velocity: 2 cm^3^ min^−1^, purity 99.99%. To reduce the siloxane blanks, a SUPELCOWAX-10 capillary column (100%—polyethyleneglycol, 10 m × 0.25 mm, 0.25 µm film thickness) was arranged instead of a CARBOWAX as tested in our previous procedure [31]. The CARBOWAX was recognized with a satisfactory chromatographic separation but the sensitivity for TMSOH determination was significantly limited (it was necessity to use an additional procedure for extraction—mixture with water 95/5; *v/v*). The auto-injection volume of the sample was 2 µL. The MS chromatograms of the biogas samples were analyzed by using the NIST 2018 MS Spectra library. The blank samples for each solvent used in the investigation were analyzed. In addition, no siloxanes were detected in these blanks. The ion fragments used for identification and quantification of VMSs as well as the retention times of standards are listed in Figure 5. The concentration of VMSs was calculated by using calibration curves. Obtained calibration curves were distinctly linear for all studied VMSs (R^2^ > 98%). The high value of the correlation coefficient illustrates the linearity of the detector within the considered concentration ranges.

## 4. Conclusions

The presented study discusses and describes the analysis of biogas from landfill, waste water treatment process, and agriculture biogas plants. Both linear and cyclic VMSs were detected and quantified in the analyzed biogas samples. It was demonstrated that the developed GC-MS method allowed for the rapid determination of cyclic and linear siloxanes in biogas samples. The conducted experiment proved, that the application of high polarity capillary columns constituted a relevant solution for the determination of siloxanes in biogas samples, as well as the gas chromatography technique coupled with mass-spectrometry detector being a viable option for siloxanes quantification. Moreover, the assessment of different sampling procedures revealed that the impinger and micro-impinger method is one of the best ways of sampling to trap siloxanes from a given gas phase. It was clearly justified, that the adsorption of siloxanes from a gas phase is strongly dependent on the polarity of the used solvent and the complexity of the biogas organic matrix has a significant influence on siloxanes determination and the validation of applied methods. The developed method for sampling and analysis was described straightforwardly and has a LoD and LoQ on the level of 0.01 and 0.04 mg/m^3^, respectively. Additionally, the developed method with the use of acetone and impinger sampling allowed for the detection of siloxanes in low levels in biogas samples. The comparison of different sampling procedures allowed for the significant reduction in the time of performance of the analysis as a result of the selection of the best solvent and sampling procedure. The obtained results confirmed that the highest concentration of siloxanes is observed in landfill biogas and the lowest in agriculture biogas. The validated method was applied for the determination of six siloxanes where the main cyclic (D5) and linear (L2) forms were found in landfill biogas. While the biogas and biomethane become more and more appreciated and utilized in Europe, its fast and reliable analysis with the application of high-polarity columns and high-polarity solvents constitute an important aspect of research for the use of biogas in modern green power development. Furthermore, the fast and simple transfer of the sampling equipment from the operated plants to a laboratory proved that the equipment used was robust and portable. Finally, the described analytical procedure represents an important contribution to the development of chromatographic analysis of siloxanes in biogas originating from different sources.

## Figures and Tables

**Table 1:** Siloxanes free energies estimated from bond energies [9].

Bond Type	Si–Si	Si–C	Si–H	C–C	C–O	C–H	Si–O
Free energies of ionization (kcal/mol)	45<	69	72	81	84	100	103

**Table 2:** Physical and chemical properties of volatile methylsiloxanes presented in biogas.

Name	Hexamethyl- Disiloxane	Octamethyl- Trisiloxane	Decamethyl- Tertasiloxane	Hexamethylcyclo- Trisiloxan	Octamethylcyclo- Tetrasiloxan	Decamethylcyclo- Pentasiloxane	Dodecamethylcyclo- Hexasiloxane	References
Abbreviation	L2	L3	L4	D3	D4	D5	D6	
Structure	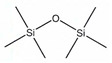	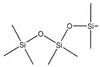	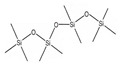	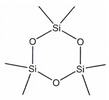	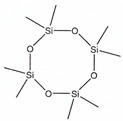	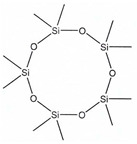	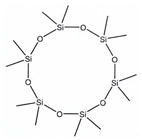	
Molecular formula	C_6_H_18_OSi_2_	C_8_H_24_O_2_Si_3_	C_10_H_30_O_3_Si_4_	C_6_H_18_O_3_Si_3_	C_8_H_24_O_4_Si_4_	C_10_H_30_O_5_Si_5_	C_12_H_36_O_6_Si_6_	
Physical properties	Liquid, colorless, odorless	Liquid, colorless, odorless	solid	Solid, white, with a hydrocarbon odor	Liquid, colorless, oily, odorless	Liquid, oily	Liquid, colorless, faint odor	[10]
Molecular weight (Da)	162.38	236.54	310.7	222.47	296.61	370.80	444.93	[10,12]
Boiling point, °C	101	153	193.95	135	175.5	210	245	[10,12]
Melting point, °C	−59	−82	no data	64	17.5	7.5	−3	[10,13]
Water solubility mg/L w 23 °C	0.93	0.034	0.006 (at 25 °C)	1.56	0.056	0.017	0.005	[10,12,14]
Vapor density (air = 1)	5.5	8.16	no data	8.00	>1	no data	no data	[10,15]
Viscosity, cP at 25 °C	0.86	1.2	1.7 (at 20 °C)	no data	2.6	no data	no data	[10,12,13]
Vapor pressure, mm Hg at 25 °C	42.2	13.90	0.43 (0.55)	3.53	1.05	0.20	0.049	[10,12,13,16]
Henry’s constant	2.4 ± 0.2, at 27 °C	121 ± 12, at 27 °C	no data	no data	24 ± 3, at 28 °C	12 ± 2, at 26 °C	5.9 ± 2.9, at 26 °C	[6,17]
Octanol/water partition coefficient (Log Pow)	4.2	4.8	no data	4.47	5.1	5.2	5.86	[13,14,18,19]
Structure	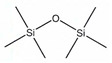	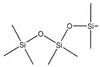	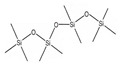	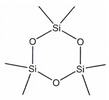	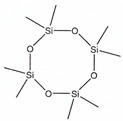	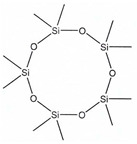	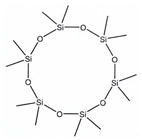	
Molecular formula	C_6_H_18_OSi_2_	C_8_H_24_O_2_Si_3_	C_10_H_30_O_3_Si_4_	C_6_H_18_O_3_Si_3_	C_8_H_24_O_4_Si_4_	C_10_H_30_O_5_Si_5_	C_12_H_36_O_6_Si_6_	[12,19,20,21,22,23,24]
Molar volume (g/cm^3^) at 20 °C	210.0	287.3	364.6	231.9	309.2	386.5	463.8	[12,19,20,21,22,23,24]
Density (g/cm^3^) at 20 °C	0.765	0.820	0.854	0.856	0.953	0.955	0.959	[12,19,20,21,22,23,24]
Critical temperature (°C)	245.15	297.95	333.35	281.05	313.35	346.05	382.25	[12,19,20,21,22,23,24]
Critical pressure (atm)	19.4	17.3	14.9	17.7	13.2	11.5	12.9	[12,19,20,21,22,23,24]
Critical volume (m^3^/kmol)	629.0	906.0	1173.7	707.1	979.0	1216.0	1493.1	[12,19,20,21,22,23,24]
References	[25]	[26]	[12,27]	[28,29]	[30,31]	[32]	[33]	

**Table 3:** Results of VMSs concentrations in analyzed biogas samples.

Siloxanes Concentrations (mg\m^3^ ± RSD)									
Method: GC-MS									
Biogas Type: VMS/Extraction Solvent	Landfill			Sewage Treatment			Agriculture		
	Acetone	Methanol	d-decane	Acetone	Methanol	d-decane	Acetone	Methanol	d-decane
Sampling: Micro-Impinger									
L2	3.4 ± 0.3	3.2 ± 0.3	0.2 ± 0.1	1.4 ± 0.1	1.0 ± 0.1	n.d	n.d	n.d	n.d
L3	0.2 ± 0.1	n.d	n.d	n.d	n.d	n.d	n.d	n.d	n.d
L4	0.1 ± 0.1	n.d	n.d	n.d	n.d	n.d	n.d	n.d	n.d
D3	9.5 ± 0.1	9.0 ± 0.1	7.0 ± 0.2	1.8 ± 0.1	1.3 ± 0.2	n.d	n.d	n.d	n.d
D4	63.5 ± 0.2	60.4 ± 0.3	59.8 ± 0.3	5.9 ± 0.1	4.5 ± 0.2	1.6 ± 0.1	0.6 ± 0.2	n.d	n.d
D5	29.7± 0.2	25.8 ± 0.3	0.2 ± 0.1	19.9 ± 0.1	17.2 ± 0.1	3.9 ± 0.1	0.9 ± 0.1	0.3 ± 0.1	n.d
D6	1.7 ± 0.1	1.3 ± 0.2	n.d	0.2 ± 0.1	0.2 ± 0.1	n.d	n.d	n.d	n.d
Sampling: Regular Impinger									
L2	3.5 ± 0.3	3.5 ± 0.3	0.3 ± 0.1	1.4 ± 0.1	1.2 ± 0.1	n.d	n.d	n.d	n.d
L3	0.2 ± 0.1	n.d	n.d	n.d	n.d	n.d	n.d	n.d	n.d
L4	0.1 ± 0.1	n.d	n.d	n.d	n.d	n.d	n.d	n.d	n.d
D3	10.6 ± 0.1	9.4 ± 0.2	7.1 ± 0.2	2.0 ± 0.1	1.6 ± 0.2	n.d	n.d	n.d	n.d
D4	65.8 ± 0.2	62.4 ± 0.2	60.0 ± 0.3	6.1 ± 0.1	4.8 ± 0.2	1.9 ± 0.1	0.8 ± 0.2	n.d	n.d
D5	30.0 ± 0.2	26.4 ± 0.2	0.3 ± 0.1	20.5 ± 0.1	17.8 ± 0.1	4.0 ± 0.1	1.1 ± 0.1	0.4 ± 0.1	n.d
D6	1.8 ± 0.1	1.4 ± 0.2	n.d	0.2 ± 0.1	0.3 ± 0.1	n.d	n.d	n.d	n.d
Sampling: AC Tube									
L2	3.2 ± 0.3	2.9 ± 0.3	n.d	1.3 ± 0.1	1.1 ± 0.1	n.d	n.d	n.d	n.d
L3	n.d	n.d	n.d	n.d	n.d	n.d	n.d	n.d	n.d
L4	n.d	n.d	n.d	n.d	n.d	n.d	n.d	n.d	n.d
D3	10.6 ± 0.1	8.1 ± 0.2	5.9 ± 0.2	1.9 ± 0.1	1.5 ± 0.1	0.6 ± 0.1	n.d	n.d	n.d
D4	65.2 ± 0.2	58.2 ± 0.2	50.3 ± 0.2	5.8 ± 0.1	5.0 ± 0.1	2.3 ± 0.2	0.7 ± 0.1	0.3 ± 0.1	n.d
D5	29.6 ± 0.2	22.4 ± 0.2	n.d	20.0 ± 0.1	18.8 ± 0.2	12.0 ± 0.2	0.3 ± 0.1	0.4 ± 0.1	n.d
D6	1.7 ± 0.1	1.0 ± 0.2	n.d	0.2 ± 0.1	0.2 ± 0.1	n.d	n.d	n.d	n.d
Sampling: TedlarBag (direct injection)									
	Landfill Biogas			Sewage Biogas			Agriculture Biogas		
L2	n.d			n.d			n.d		
L3	n.d			n.d			n.d		
L4	n.d			n.d			n.d		
D3	5.9 ± 0.3			0.7 ± 0.2			n.d		
D4	49.1 ± 0.2			2.6 ± 0.2			n.d		
D5	16.6 ± 0.1			11.6 ± 0.3			n.d		
D6	n.d			n.d			n.d		

**Table 4:** GC-MS parameters of calibration curves and validation.

System: Varian 3800, CTC, Saturn IT2200/GC-MS						
VMS	Calibration Curves Parameters Impinger Method (Solvent: Acetone)					Range of Linearity (mg/m^3^)
	y = ax + b					
	a ± RSD	B ± RSD	LoD	LoQ	R^2^	
			(mg/m^3^)			
Landfill Biogas—Direct from TedlarBag						
L2	219.16 ± 19.08	−425.05 ± 12.52	0.08	0.10	0.989	0.14–5.00
L3	409.12 ± 10.06	−504.13 ± 20.86	0.06	0.11	0.986	0.12–2.83
L4	502.36 ± 14.69	−917.23 ± 14.09	0.09	0.11	0.985	0.14–2.00
D3	409.52 ± 10.67	−103.81 ± 20.06	0.03	0.06	0.992	0.10–30.62
D4	301.08 ± 17.04	−612.00 ±103.04	0.02	0.06	0.997	0.10–70.06
D5	326.06 ± 10.27	−5490.23 ± 103.16	0.02	0.04	0.991	0.10–30.60
D6	336.69 ± 11.15	452.20 ± 50.01	0.04	0.09	0.992	0.11–2.06
Sewage Biogas—AC Tube (with extraction, solvent: acetone/methanol)						
L2	286.56 ± 12.03	−619.06 ± 13.15	0.04	0.08	0.994	0.12–5.06
L3	319.25 ± 8.12	−114.96 ± 2.06	0.04	0.08	0.993	0.12–3.08
L4	293.22 ± 9.06	−63.11 ± 19.85	0.04	0.06	0.991	0.14–1.06
D3	366.06 ± 13.57	−106.25 ± 8.22	0.03	0.05	0.992	0.10–40.09
D4	488.14 ± 19.03	99.11 ± 2.98	0.01	0.04	0.991	0.11–80.84
D5	477.02 ± 19.06	−63.00 ± 9.14	0.01	0.05	0.995	0.11–40.09
D6	611.42 ± 10.04	62.22 ± 14.93	0.01	0.04	0.992	0.11–3.95
Agriculture Gas (Impinger Method)						
L2	504.55 ± 19.06	−247.00 ± 15.03	0.03	0.05	0.997	0.11–10.52
L3	18.06 ± 2.06	−85.09 ± 6.16	0.02	0.06	0.994	0.12–10.06
L4	1274.51 ± 15.23	−52.85 ± 14.00	0.02	0.05	0.998	0.10–25.16
D3	506.76 ± 3.85	−85.04 ± 13.02	0.02	0.05	0.997	0.10–50.62
D4	607.55 ± 14.06	106.07 ± 26.33	0.01	0.04	0.997	0.10–100.74
D5	1019.00 ± 52.03	−69.04 ± 8.04	0.01	0.05	0.996	0.11–50.20
D6	704.03 ± 23.06	106.08 ± 25.88	0.01	0.04	0.996	0.14–10.60

## Data Availability

The data used to support the findings of this study are available from the corresponding author upon request.

## Sample Availability

Samples of the analyzed biogases were spent.
